# MR imaging characteristics of uveal melanoma with histopathological validation

**DOI:** 10.1007/s00234-021-02825-5

**Published:** 2021-10-31

**Authors:** Teresa A. Ferreira, Myriam G. Jaarsma-Coes, Marina Marinkovic, Berit Verbist, Robert M. Verdijk, Martine J. Jager, Gregorius P. M. Luyten, Jan-Willem M. Beenakker

**Affiliations:** 1 Department of Radiology, Leiden University Medical Center, Albinusdreef 2, 2333 ZA Leiden, The Netherlands; 2 Department of Ophthalmology, Leiden University Medical Center, Albinusdreef 2, 2333 ZA Leiden, The Netherlands; 3 Department of Pathology, Leiden University Medical Center, Albinusdreef 2, 2333 ZA Leiden, The Netherlands; 4 Department of Pathology, Section Ophthalmic Pathology, Erasmus MC University Medical Center, Burgemeester Oudlaan 50, 3062 PA Rotterdam, The Netherlands

**Keywords:** Uveal melanoma, Magnetic resonance imaging, Ultrasound, Histopathology, Ocular oncology

## Abstract

**Purpose:**

To evaluate the magnetic resonance imaging (MRI) characteristics of uveal melanoma (UM), to compare them with fundoscopy and ultrasound (US), and to validate them with histopathology.

**Methods:**

MR images from 42 UM were compared with US and fundoscopy, and on 14 enucleated cases with histopathology.

**Results:**

A significant relationship between the signal intensity on T1 and pigmentation on histopathology was found (*p*=0.024). T1 hyperintense UM were always moderately or strongly pigmented on histopathology, while T1-hypointense UM were either pigmented or non-pigmented. Mean apparent diffusion coefficient (ADC) of the UM was 1.16 ± 0.26 × 10^−3^ mm^2^/s. Two-thirds of the UM had a wash-out and the remaining a plateau perfusion time-intensity curve (TIC). MRI was limited in evaluating the basal diameter of flat tumors. US tends to show larger tumor prominence (0.5mm larger, *p*=0.008) and largest basal diameter (1.4mm larger, *p*<0.001). MRI was good in diagnosing ciliary body involvement, extrascleral extension, and optic nerve invasion, but limited on identifying scleral invasion. An increase of tumor prominence was associated with lower ADC values (*p*=0.030) and favored a wash-out TIC (*p*=0.028). An increase of tumor ADC correlated with a plateau TIC (*p*=0.011).

**Conclusions:**

The anatomical and functional MRI characteristics of UM were comprehensively assessed. Knowing the MRI characteristics of UM is important in order to confirm the diagnosis and to differentiate UM from other intra-ocular lesions and because it has implications for treatment planning. MRI is a good technique to evaluate UM, being only limited in case of flat tumors or on identifying scleral invasion.

**Supplementary Information:**

The online version contains supplementary material available at 10.1007/s00234-021-02825-5.

## Introduction

Uveal melanoma (UM) is the most common primary intraocular malignancy in adults [1–4]. UM is very different from cutaneous melanoma in terms of survival and genetic profile and in the lack of effective therapies to prevent metastatic disease [5–7]. In the past, enucleation was the main treatment, but over the last decades, various eye- and vision-saving treatments have become available, including episcleral brachytherapy, proton beam radiotherapy, and stereotactic radiotherapy [2, 3, 8].

Ultrasound (US), fundoscopy, and fluorescein angiography are the most frequently used techniques to evaluate UM at the diagnosis, for pre-treatment planning, and for follow-up after radiotherapy [3, 4]. However, the diagnosis with these conventional ophthalmic imaging modalities is difficult in smaller uveal melanomas/melanocytic lesions, in atypical tumors, in lesions behind the iris, and in case of opacification of the ocular media. Furthermore, US has limitations in pre-treatment planning of UM and during follow-up, being only able to evaluate dimensional changes of the lesion. Another option for imaging the globe is magnetic resonance imaging (MRI), which has sometimes been challenging because of eye motion and/or susceptibility artifacts [8–10]. However, recent developments on MRI allow good quality images [8]. Moreover, with a high soft tissue contrast and spatial resolution, the possibility of generating 3D volumetric and functional images, and the possibility of evaluating UM in eyes of vitrectomized patients with a SiOil tamponade, MRI seems to be of added value in comparison with US [8, 11–13].

In this study, we evaluate the use of MRI in a series of primary UM. Firstly, we will provide a complete description of the radiological characteristics of UM, which can aid in using MRI to differentiate UM from other intra-ocular lesions, as for UM biopsies are generally not performed to confirm the diagnosis. This evaluation will not only include anatomical parameters, such as signal intensities on T1- and T2-weighted images (WI), but also include functional parameters, such as the apparent diffusion coefficient (ADC) and quantifiable perfusion characteristics. Secondly, we will compare clinical parameters related to treatment and/or prognostication, such as tumor dimensions, pigmentation, and involvement of nearby structures, between MRI and conventional ophthalmic techniques, including fundoscopy and US. These findings will be validated with histopathology when available. Finally, attention will be given to potential MRI prognostic markers, which would help to identify high-risk UM.

## Methods

Forty-two patients with the diagnosis of primary UM were evaluated. The first cohort consisted of thirty consecutive patients, who were prospectively evaluated at the Leiden University Medical Center (LUMC), as part of a single-institution prospective study, carried out according to the Declaration of Helsinki. Following approval of the protocol by the local Medical Ethical Committee (METC P16.186), informed consent was obtained from all participants. The second group consisted of twelve patients, whose eyes were scanned for a clinical reason and that were retrospectively evaluated, with permission from the local Medical Ethical Committee. This group included one case treated with ruthenium brachytherapy, four cases that received proton beam therapy, and seven cases that underwent enucleation.

All 42 patients were examined by an ocular oncologist, and the final diagnosis was made on the basis of fundoscopic, fluorescein angiographic, and ultrasonographic findings, prior to the MRI examination.

The mean age of all subjects was 62 years (range 24–90) and 69% were male. In 61% of the patients, the lesion was localized in the right eye. The clinical American Joint Committee of Cancer T-Stage of the UM was T1 in 29%, T2 in 31%, T3 in 31%, and T4 in 9%. Regarding treatment, 45% of the cases received ruthenium brachytherapy, 21% proton beam therapy, and 33% were enucleated. In one patient, the diagnosis was not clear, and, prior to the MRI, a biopsy was performed, disclosing a UM. In all 14 patients who underwent enucleation histopathology confirmed the clinical diagnosis of UM (Supplementary Table 1).

**Fig. 1:**
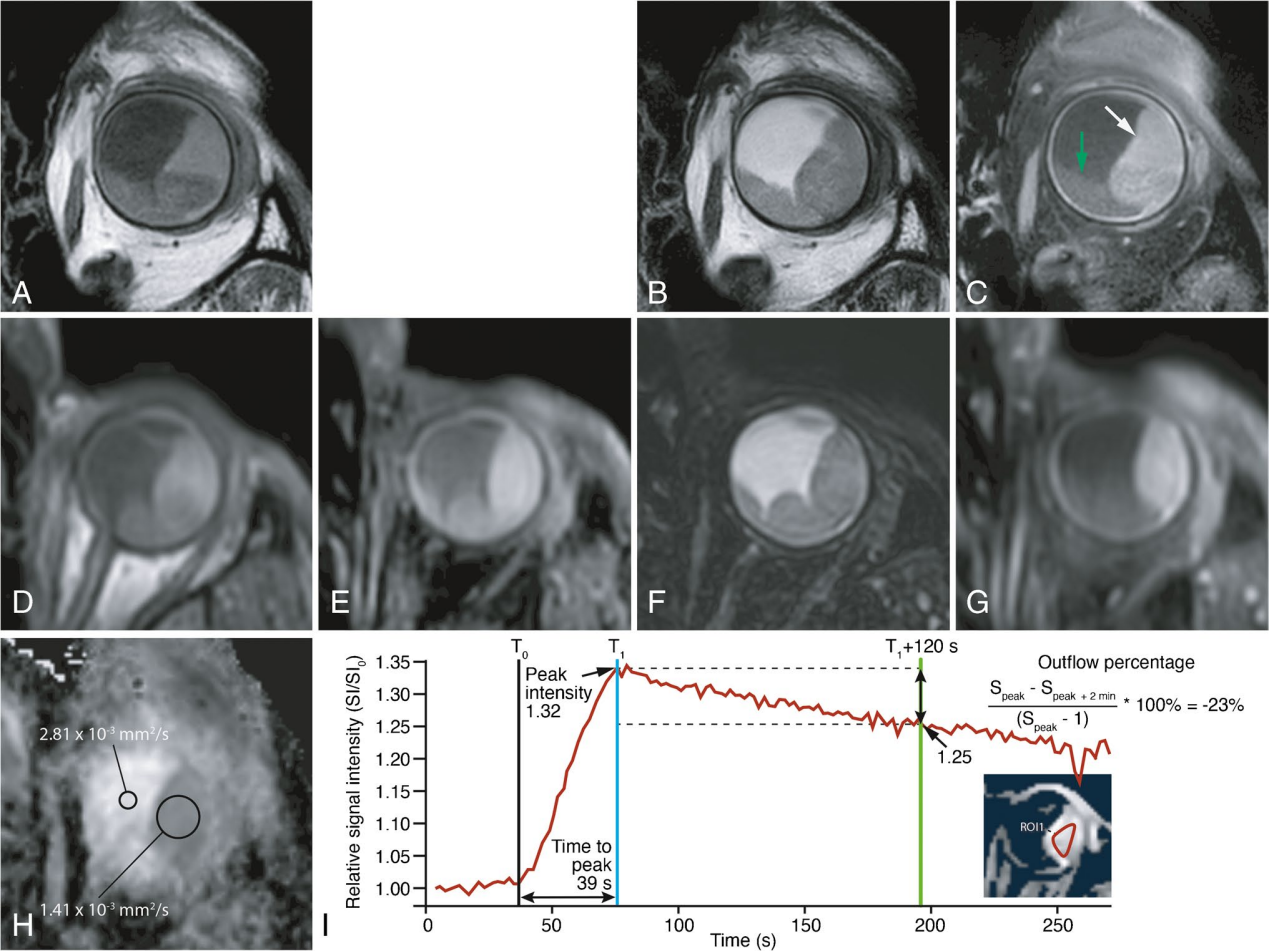
MRI ocular protocol. Uveal melanoma of the left eye (white arrow) with associated retinal detachment (green arrow). ADC of 1.4 × 10^−3^ mm^2^/s. Wash-out time-intensity curve at DCE. **A** MS T1. **B** MS T2. **C** MS contrast-enhanced T1 with fat signal suppression. **D** 3D TSE T1. **E** 3D TSE T1 with fat signal suppression. **F** 3D TSE T2 with fat signal suppression. **G** 3D TSE contrast-enhanced T1 with fat signal suppression. **H** ADC. **I** Quantitative evaluation of the DCE. Black line—arrival time (T0) = timepoint at which the lesion starts to enhance, determined manually. Blue line—corresponds approximately to the peak time (T1) = first timepoint when the lesion reached 95% of its maximum intensity, determined automatically and corresponding to the timepoint at which peak intensity (PI) is calculated. Time to peak (TTP) (s) = T1-T0. Green line—outflow percentage at 2 min (OP2,%) = percentage of signal intensity loss at 2 min compared to the intensity at the peak time. Notice the wash-out time-intensity curve. SI, signal intensity at every timepoint. SI_0_, signal intensity at timepoint zero

All patients underwent a 3T MRI (wide bore Ingenia 3T, Philips Healthcare, Best, The Netherlands), using the ocular protocol that we previously developed [8], with minor adjustments: a higher resolution of the 3D TSE T1 sequences, diffusion weighted imaging (DWI) with *B* values only of 0 and 800 s/mm^2^, and a higher flip angle of the dynamic scan (Table 1; Figure 1).

**Table 1:** MRI scans’ parameters including both anatomical and functional sequences. The MS and DWI sequences are acquired perpendicular to the main axis of the tumor. The 3D TSE and DCE sequences are acquired on the axial plane non-angulated. During the DCE scan, intravenous administration of 0.1 mmol/kg gadoterate meglumine (gd-DOTA, DOTAREM, Guerbet, Roissy CdG Cedex, France) is administered and afterwards, the contrast-enhanced (Gd) scans are acquired

Purpose	Scan name	Voxel size (mm^3^)	Echo train length	TE(ms)/TR(ms)/Flip or ref. angle (deg)	Fat supr.	Scan time (mm:ss)	Additional parameters
3D measurements	**3D TSE T1**	0.8×0.8×0.8	20	26/400/90	-	02:07	
	**3D TSE T1 SPIR**	0.8×0.8×0.8	20	26/400/90	SPIR	02:07	
	**3D TSE T2 SPIR**	0.8×0.8×0.8	117	305/2500/35	SPIR	02:58	
	**3D TSE T1 SPIR Gd**	0.8×0.8×0.8	20	26/400/90	SPIR	02:07	
Tumor origin and extension	**MS TSE T1**	0.5×0.5×2.0	6	8/718/180	-	01:16	
	**MS TSE T2**	0.4×0.4×2.0	17	90/1331/120	-	01:25	
	**MS TSE T1 SPIR Gd**	0.5×0.5×2.0	6	80/764/180	SPIR	01:16	
Functional scans	**DWI (TSE)**	1.25×1.4×2.4	Single shot	50/1555/50	SPIR	01:33	*B*=0.800 s/mm^2^
	**DCE**	1.25×1.5×1.5		2.3/4.5/13	Proset 11	04:20	2 s/dynamic

Tumor origin (choroid, ciliary body, or iris) was assessed on MRI. The presence of a mushroom configuration was evaluated on MRI; when histopathology was available, it was correlated with rupture of Bruch’s membrane. The signal intensity of UM on MRI was assessed and it was evaluated whether it reflects UM pigmentation, by comparing it both to fundoscopy and histopathology. Tumor signal intensity on T1- and T2-WI was classified as hyper-, iso-, or hypointense. When UM were compared to the vitreous on MRI, all were hyperintense on T1- and hypointense on T2-WI, showing the vitreous to be an unsuitable reference for their signal intensity. Better differentiation of signal intensities was obtained when using the signal intensity of the choroid as reference on T1- and of the eye muscles on T2-WI. Tumor pigmentation on fundoscopy was categorized as pigmented or non-pigmented. Tumor pigmentation on histopathology was classified according to their macroscopic color as seen in several cuts through the UM: white, no pigmentation; yellow/gray, slight pigmentation; brown, moderate pigmentation; black, strong pigmentation. We compared UM signal intensity on T1- and T2-WI and clinical pigmentation as seen on fundoscopy in the group of homogeneous or minimally heterogeneous UM (*n*=36). The six bipartite UM were excluded in this evaluation. We furthermore compared UM signal intensity on T1- and T2-WI with pigmentation on histopathology in the group of the 14 enucleated eyes. On the four enucleated eyes with bipartite UM, both components were separately checked, accounting for a total of 18 evaluable lesions. For the ADC measurements, one representative region of interest (ROI) was drawn by the same neuroradiologist, excluding the tumor edge and potential necrotic parts, and one reference ROI was drawn in the vitreous. The tumor perfusion characteristics—arrival time (T0) (s), time to peak (TTP) (s), peak intensity (PI), outflow percentage at 2 min (OP2,%), and the type of time-intensity curve (TIC)—were evaluated in 3D, all results corresponding therefore to the average from the whole UM. Additionally, the type of TIC was also evaluated in 2D: in homogeneous tumors from a representative 2D image, and in bipartite tumors from both tumor components. The type of TIC was classified according to Yuan et al. as persistent, plateau, or wash-out pattern [14]. However, to limit the effect of potential eye movement, the outflow was evaluated at 2 min, instead of the 5 min, being the plateau and wash-out patterns defined as a final intensity at 2 min of 95–100% and below 95% of the peak intensity, respectively (Figure 1) [14]. Tumor dimensions and tumor extension were evaluated on MRI and US and validated with histopathology when available. The presence of retinal detachment (RD) was evaluated with MRI and US. Finally, on the enucleated eyes, the presence of extracellular matrix patterns (defined as three adjacent full loops) and of monosomy 3 in the tumor was checked (Supplementary Table 2).

## Results

### Origin and shape

**Fig. 2:**
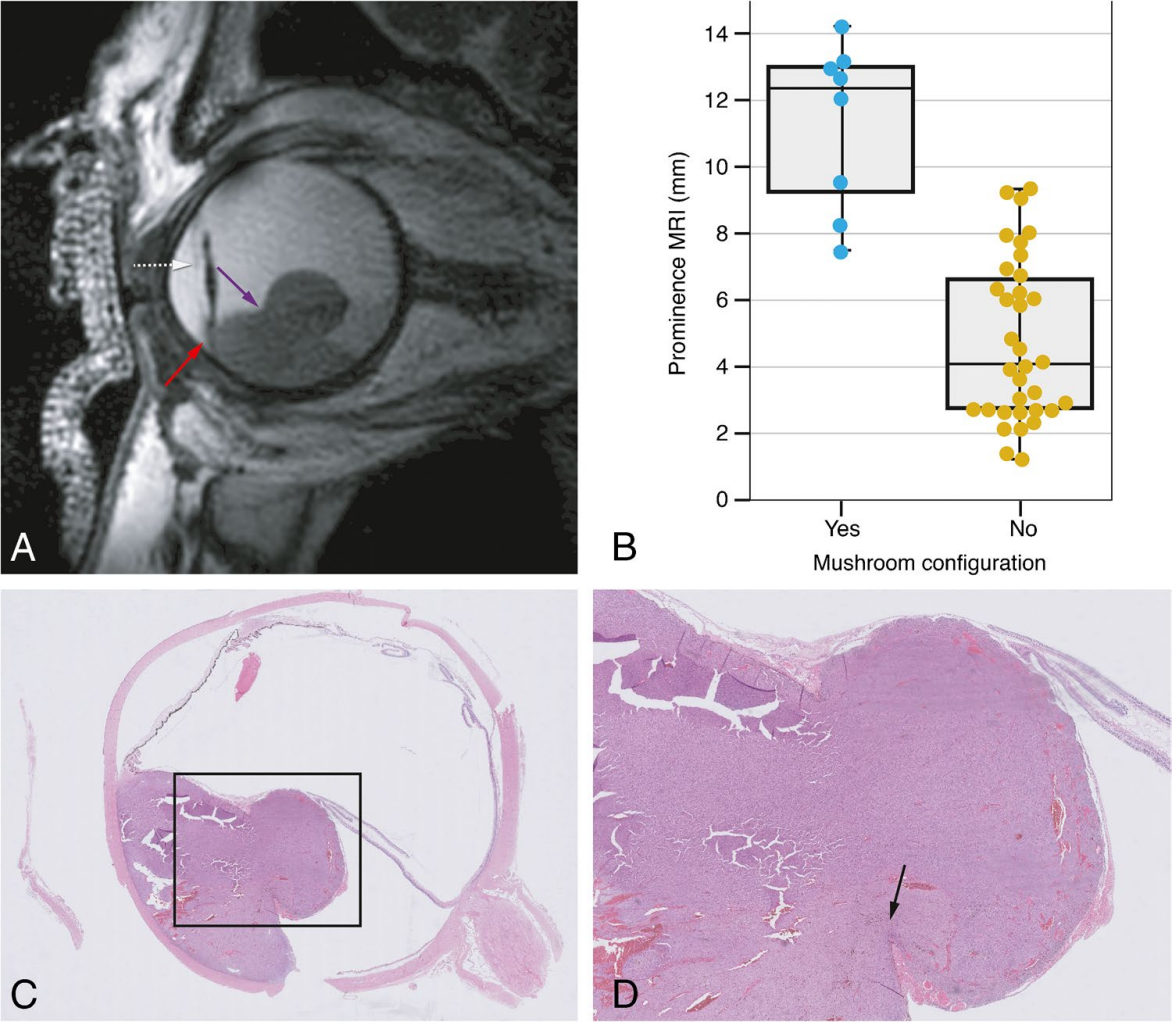
MR image of a mushroom configuration, its corresponding histopathological image, and the comparison of tumor prominence between uveal melanomas with and without a mushroom shape. **A** MRI with sagittal MS TSE T2-WI showing a UM with a mushroom configuration (purple arrow). There is invasion of the ciliary body and iris (red arrow). Notice that the iris can be clearly identified (white dashed arrow) and that the patient has an intraocular lens. **B** Boxplot to illustrate the relation between the presence of a mushroom configuration and tumor prominence (*n*=42). It is evident that a mushroom configuration is more present in tumors with larger prominences, with five patients having a tumor prominence of 12 mm or more. **C**–**D** Histopathologic examination hematoxylin and eosin stain (H&E). Notice the rupture of the Bruch membrane in the region of the neck of the mushroom (black arrow)

In 57% of the cases, the UM originated in the choroid, in 38% in either the choroid or ciliary body, and in 5% (*n*=2) in the iris. In five patients (12 %), a mushroom configuration was seen on MRI. All these five patients have undergone an enucleation and Bruch’s membrane rupture was confirmed in all. Histopathology did reveal rupture of Bruch’s membrane in three additional melanomas which had not been classified as mushroom configuration on MRI. However, retrospectively and mainly with the help of multiplanar reconstructions, a mushroom configuration was found on MRI as well. As expected, a mushroom configuration was more prevalent in larger tumors (Figure 2).

### Signal intensity

**Fig. 3:**
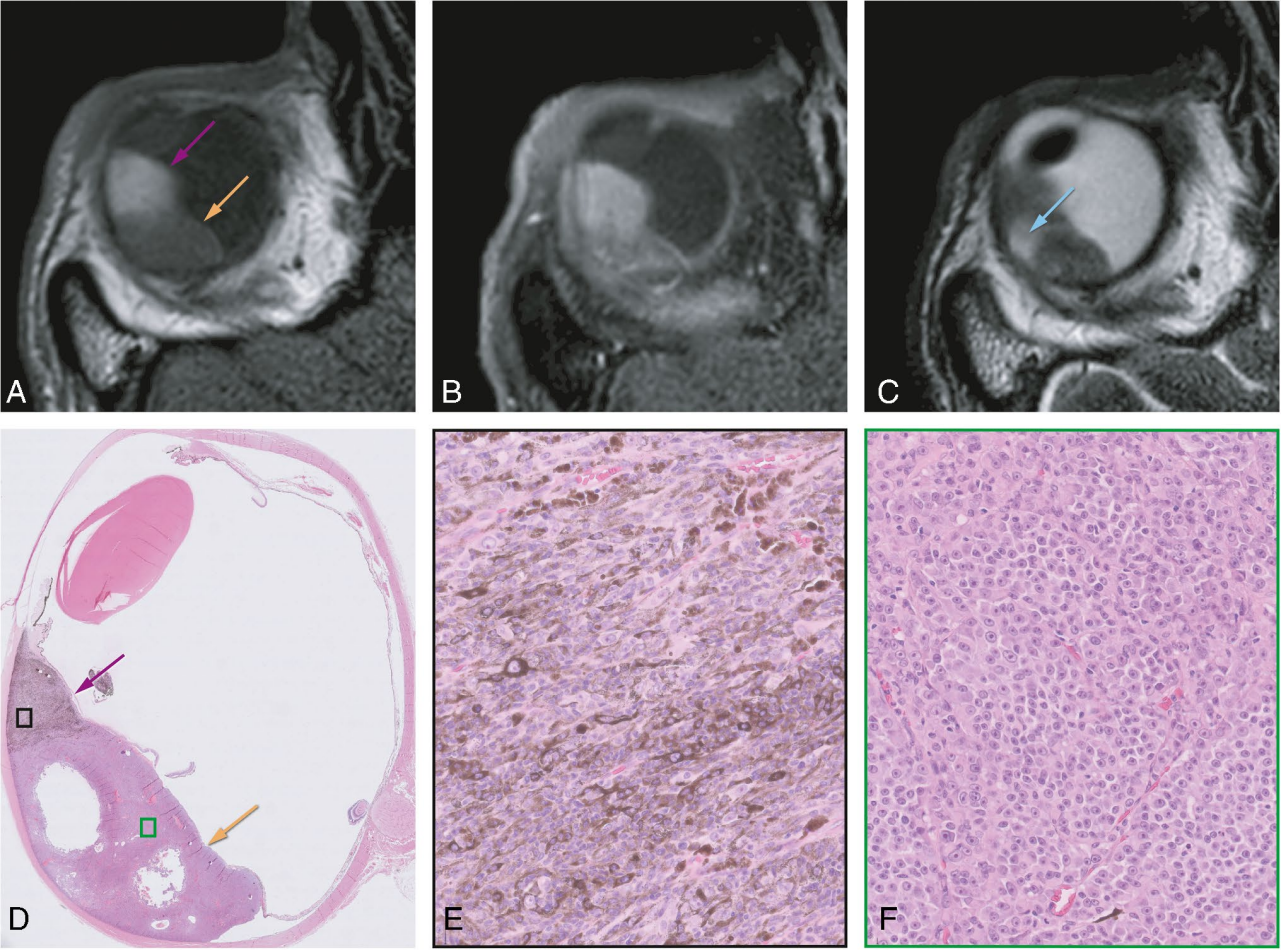
Bipartite uveal melanoma with a good correspondence between signal intensity on T1 and pigmentation on histopathology. **A**–**C** MRI with axials MS TSE T1-WI (**A**), contrast-enhanced T1-WI with fat signal suppression (**B**), and T2-WI (**C**). **D**–**F** Histopathologic examination H&E. Bipartite UM of the right eye, with one component which is hyperintense on T1 and on histopathological examination strongly pigmented (pink arrows and **E**), and a second component which is hypointense on T1 and on histopathological examination non-pigmented (orange arrows and **F**). Notice that on T2, these two different components are not differentiated, but a small hyperintense area (blue arrow) is seen corresponding to a cystic necrotic area depicted on the histopathological examination. Notice also the presence of both epithelioid and spindle cells on the pigmented component (**E**), while the non-pigmented part only harbors epithelioid cells (**F**)

Tables 2 and 3 show the comparison between signal intensity on T1- and T2-WI and fundoscopy and histopathology, respectively. A significant relationship between the signal intensity on T1-WI and pigmentation on histopathology was found (Kruskal-Wallis test; chi-square=7.45; *p*=0.024). No significant relationship between the signal intensity on T2-WI and pigmentation on histopathology was found (Kruskal-Wallis test; chi-square=2.03; *p*=0.36), neither between the signal intensity on T1-WI and pigmentation on fundoscopy (chi-square test; chi-square=2.22; *p*=0.33) nor between the signal intensity on T2-WI and pigmentation on fundoscopy (chi-square test; chi-square=0.583; *p*=0.75) (Figure 3).

**Table 2:** Contingency table comparing the signal intensity of the homogeneous UM on T1- and T2-WI with the pigmentation at fundoscopy

		Pigmentation fundoscopy		**Total**
		Yes	No	
Signal intensity T1-WI	Hyperintense	13	1	14 (39%)
	Isointense	11	4	15 (42%)
	Hypointense	5	2	7 (19%)
Signal intensity T2-WI	Hyperintense	22	6	28 (78%)
	Isointense	5	1	6 (17%)
	Hypointense	2	0	2 (5%)
Total		29 (81%)	7 (19%)	36 (100%)

**Table 3:** Contingency table comparing the signal intensity of the UM on T1- and T2-WI with the pigmentation at histopathology

		Pigmentation histopathology				Total
		Strong	Moderate	Slight	No	
Signal intensity T1-WI	Hyperintense	4	3	0	0	7 (39%)
	Isointense		2	2	1	5 (28%)
	Hypointense	1	1	1	3	6 (33%)
Signal intensity T2-WI	Hyperintense	2	4	3	3	12 (67%)
	Isointense	2	0	0	1	3 (17%)
	Hypointense	1	2	0	0	3 (17%)
Total		5 (28%)	6 (33%)	3 (17%)	4 (22%)	18 (100%)

### DWI

**Fig. 4:**
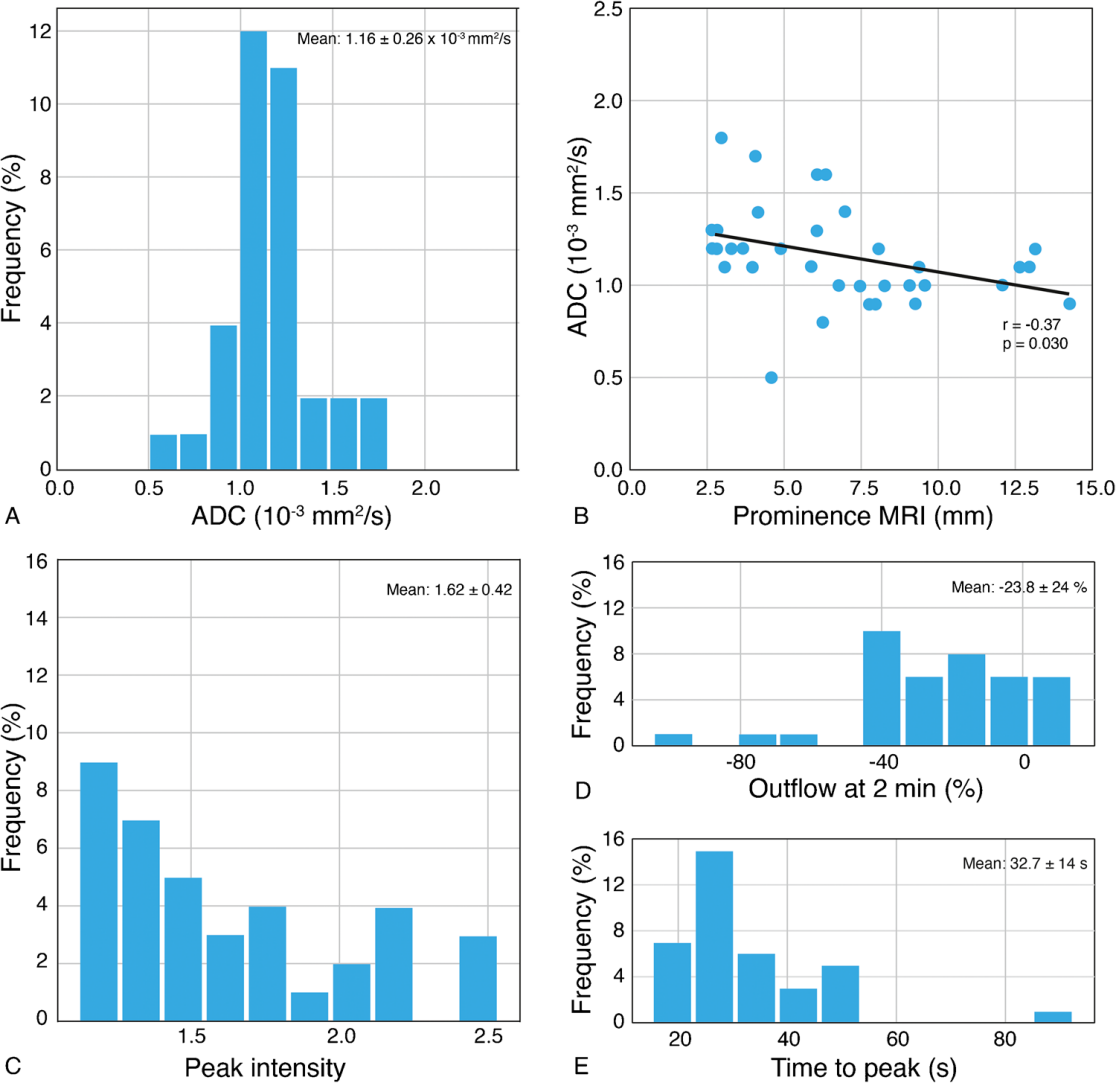
ADC and DCE quantitative results. **A** Histogram with ADC results of the UM (*n*=35). **B** Scatterplot ADC value versus tumor prominence (*n*=35), showing that larger tumors tend to have lower ADC values (*r* = −0.367; *p*=0.03). **C** Histogram with peak intensity results. **D** Histogram with outflow percentage 2 min results. **E** Histogram with time to peak results

Seven UM were excluded from ADC evaluation, due to unreliable measurement in tumors with a prominence less than 2.5 mm, or due to technical problems. The distribution of UM ADCs (mean: 1.16 ± 0.26 × 10^−3^ mm^2^/s) is shown in Figure 4A. Mean ADC of the vitreous was 3.03 ± 0.17 × 10^−3^ mm^2^/s.

### Perfusion weighted imaging (PWI)

Five UM were excluded from the 3D evaluation and three UM were also excluded from the 2D evaluation, due to eye motion in tumors with a prominence of 3.5 mm or less, or due to technical problems. The perfusion quantitative results are shown in Figure 4C–Eand in Supplementary Figure 1.

The 3D evaluation showed in 65% of the cases a wash-out TIC and in 35% a plateau TIC. The 2D evaluation showed in 64% a wash-out TIC, at 28% a plateau TIC, and at 8% both a wash-out and a plateau TIC. In seven patients (19%), the qualitative evaluation of the time-intensity curves differed between the 3D and 2D evaluations.

### Dimensions

**Fig. 5:**
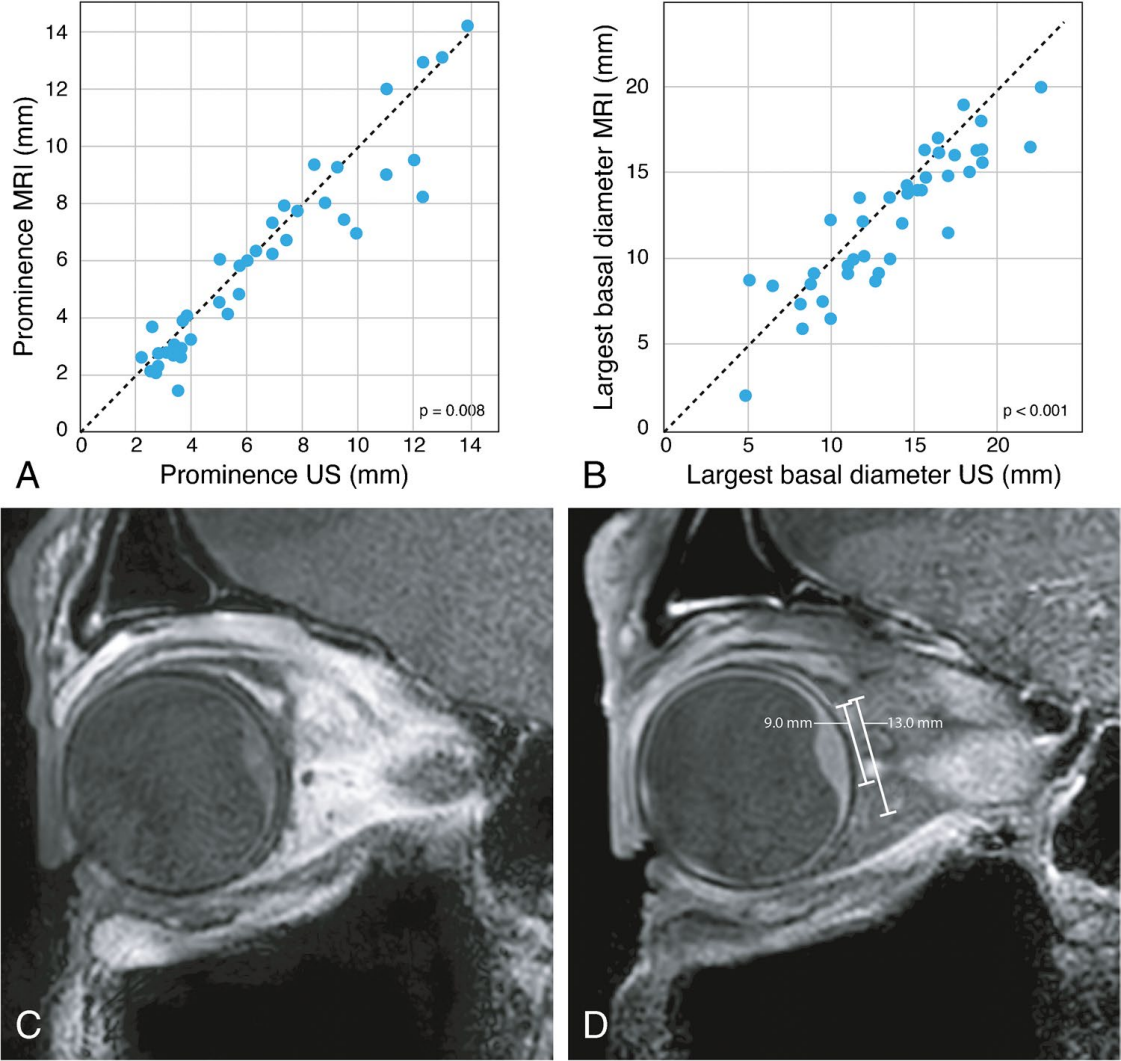
Comparison of tumor prominence and largest basal diameter by MRI and by US measurement with a good correlation found for both dimensions. MRI limitations on measuring the largest basal diameter in flat uveal melanomas. **A** Scatterplot tumor prominence US versus MRI. **B** Scatterplot tumor largest basal diameter US versus MRI. **C**–**D** MRI with sagittals MS TSE T1-WI (**C**) and contrast-enhanced T1-WI with fat signal suppression (**D**) showing different tumor basal diameters taking the peripheral flat tumor components into account or not. On MRI, a basal diameter of 9 mm was erroneously measured, while histopathology showed 14 mm. Retrospectively and taking the peripheral components of the tumor into account, 13 mm was obtained

A paired *t* test of the mean difference showed that both the tumor prominence (TP) (measured including the scleral thickness) and tumor largest basal diameter (LBD) were significantly higher on US than on MRI (mean TP 6.43 mm and 5.94 mm; SD 3.46 and 3.37; *p*=0.008) (mean LBD 13.70 mm and 12.31 mm; SD 4.38 and 4.03; *p*<0.001). When compared to histopathology, both dimensions were smaller on histopathology than on MRI in 11 out of 14 cases, which is to be expected due to shrinkage. There was one case however where the LBD was 5 mm larger on histopathology than on MRI. This likely was due to a peripheral flat component of the tumor (Figure 5).

### Ciliary body involvement

Histopathology and MRI were consistent regarding ciliary body involvement: 57% of the enucleated eyes showed ciliary body involvement on MRI as well as in histopathology (Figure 2).

### Scleral invasion

**Fig. 6:**
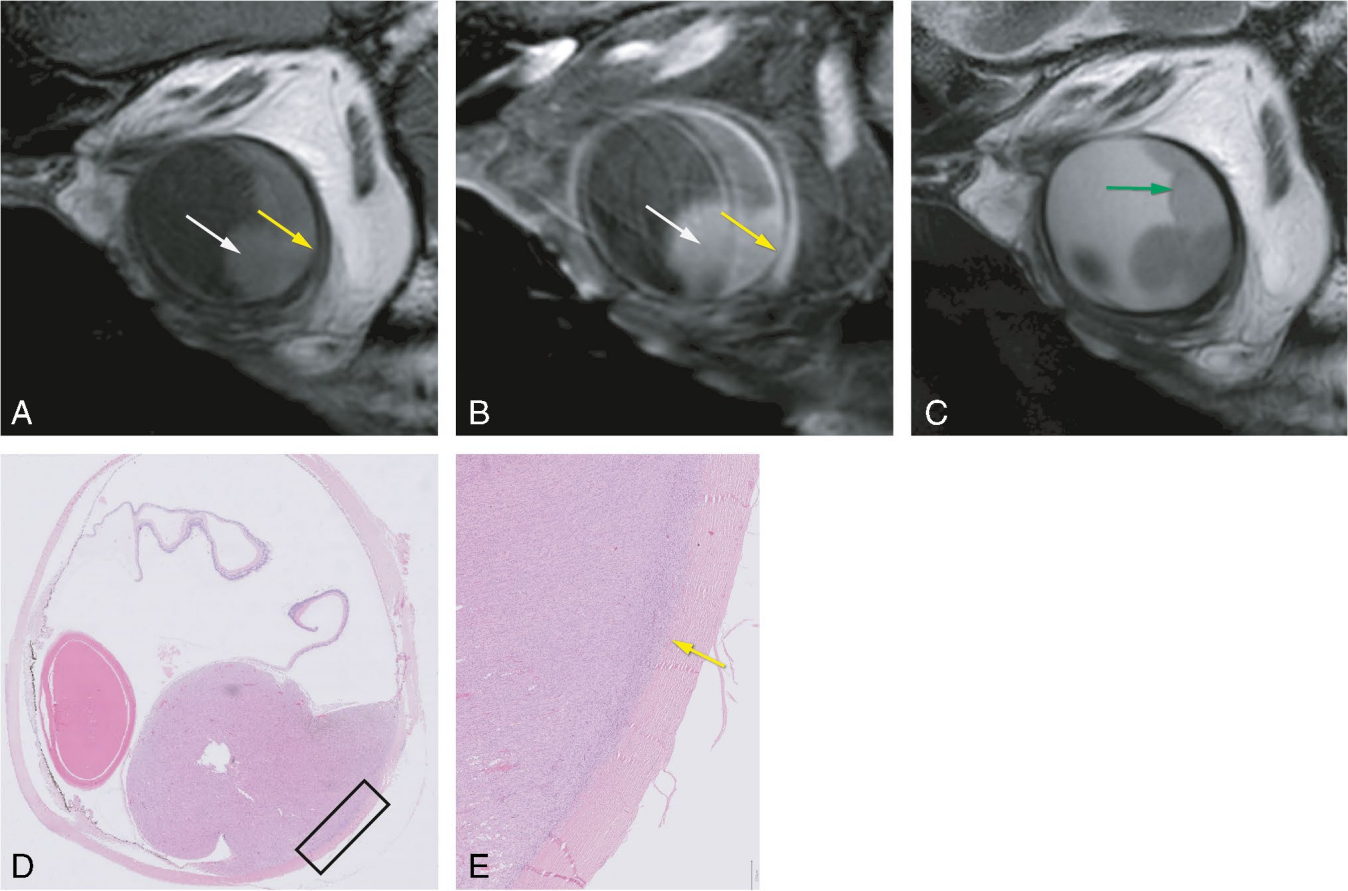
MR and histopathological images of a uveal melanoma with scleral invasion. **A**–**C** MRI with sagittals MS TSE T1 (**A**), contrast-enhanced T1-WI with fat signal suppression (**B**), and T2-WI (**C**). Uveal melanoma (white arrow) with scleral invasion. The scleral invasion (yellow arrow) is seen as sclera enhancement and irregularity. Associated retinal detachment (green arrow). **D**–**E** Histopathologic examination H&E with corresponding findings of scleral invasion (yellow arrow)

Although not seen on MRI in any of the 42 patients, scleral invasion was present in the histopathologic sections in 10 of the 14 enucleated eyes (71%). Retrospectively, minimal signs of scleral invasion could be observed on MRI in two patients (Figure 6), with an irregular inner contour and slight enhancement of the sclera.

### Extrascleral extension

**Fig. 7:**
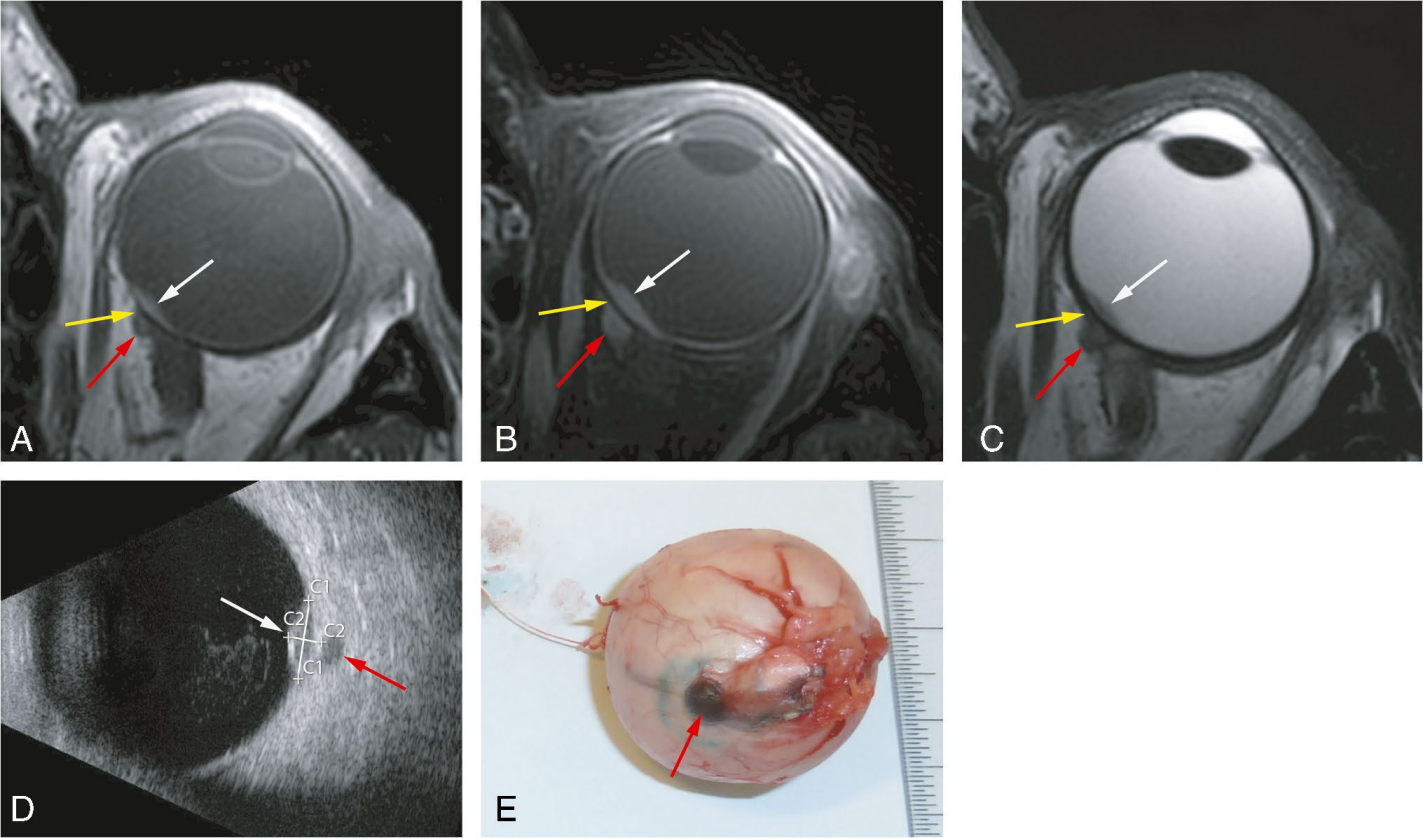
MR and grossing images of a uveal melanoma with extrascleral extension. **A**–**C** MRI with axials MS TSE T1-WI (**A**), contrast-enhanced T1-WI with fat signal suppression (**B**), and T2-WI (**C**). Small lentiform shape UM of the left eye (white arrow), with the extrascleral extension (red arrow) having, on all sequences, a signal intensity similar to its intraocular component. Notice the sclera, between the intra- and extraocular tumor components, with a normal aspect (yellow arrow). **D** B-scan ultrasound showing the small UM (white arrow) and also clearly the extrascleral extension (red arrow). **E** Enucleated eye at grossing with visible scleral and extrascleral extension (red arrow)

MRI depicted extrascleral extension in three patients (7%), which was confirmed during surgical placement of tantalum markers (one patient) or on histopathology after enucleation (two eyes). In two of these three patients, the extrascleral extension was also seen with B-Scan US. Of the other 12 enucleated eyes, one case of extrascleral extension was diagnosed on histopathology, which had been missed on MRI: this concerned a small anteriorly located lesion (maximal diameter 1.1 mm), which had already been noted clinically during slit lamp examination. Retrospectively, it was visible on MRI (Figure 7).

### Optic nerve invasion

**Fig. 8:**
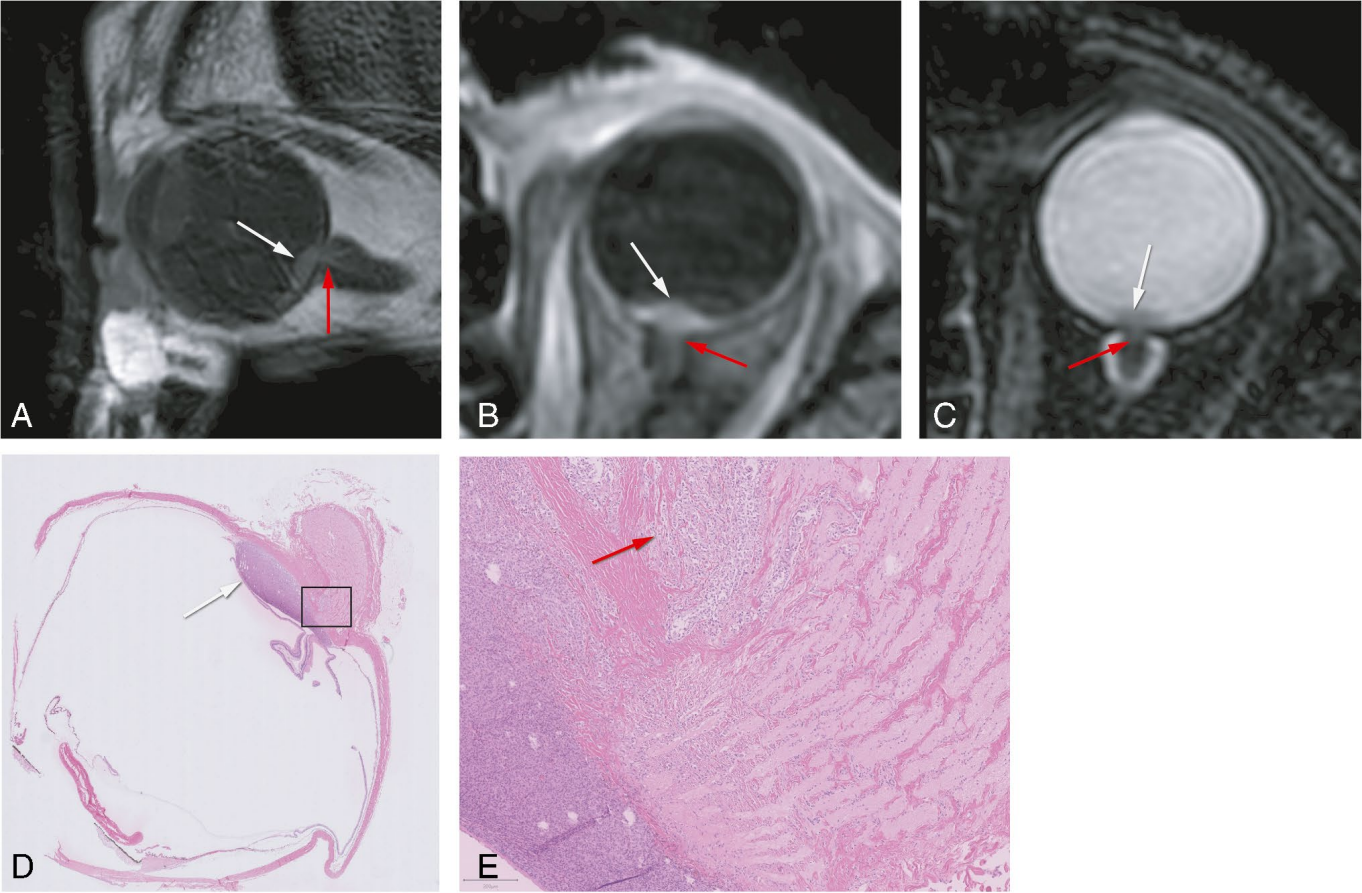
MR and histopathological images of a uveal melanoma with optic nerve invasion. **A**–**C** MRI with sagittal MS TSE T1-WI (**A**) and axials 3D TSE contrast-enhanced T1-WI with fat signal suppression (**B**) and T2-WI with fat signal suppression (**C**) showing a small lentiform shape UM of the left eye (white arrow) with minimal invasion of the optic nerve (red arrow). **D**–**E** Histopathologic examination H&E showing the UM (white arrow) with minimal invasion of the optic nerve (red arrow)

On MRI, the tumor approached the optic nerve head in fifteen patients (36%). Invasion of the postlaminar aspect of the optic nerve was seen in one patient (2%), and this was confirmed histopathologically. The invasion had not been visualized with US (Figure 8). In all other patients where histopathology was available (*n*=13), the non-involvement of the optic nerve on MRI was confirmed histopathologically.

### Retinal detachment

RD was present in 62% of the UM-containing eyes on MRI, while on US, it was seen in 38%: a difference of 24%. There were no cases in which US noticed RD that was not seen on MRI. RD occurred more frequently in tumors with a large prominence and in tumors with a large basal diameter (Supplementary Figure 2).

### Extracellular matrix patterns (loops) and Monosomy 3

Ten UM (72%) showed loops, while three UM (21%) had no loops. In one patient (7%), the presence of loops could not be evaluated. All ten tumors with loops had monosomy 3 and all three tumors without loops had disomy 3.

### Relation between prognostic markers and functional MRI characteristics

A significant correlation between tumor ADC and TP on MRI was found using the Pearson correlation coefficient (*r* = −0.37; *p*=0.030), with tumors with larger TP tending to have lower ADC values (Figure 4B). A significant relation between TIC type and TP on MRI was found using logistic regression, showing that an increase of the TP favors a wash-out TIC (OR=1.37, *p*=0.028). An increase of tumor ADC significantly correlated with a plateau TIC using logistic regression (OR=0.001, *p*=0.011). No significant relationship between TIC type and LBD on MRI was found using logistic regression (OR=1.23, *p*=0.055).

**Fig. 9:**
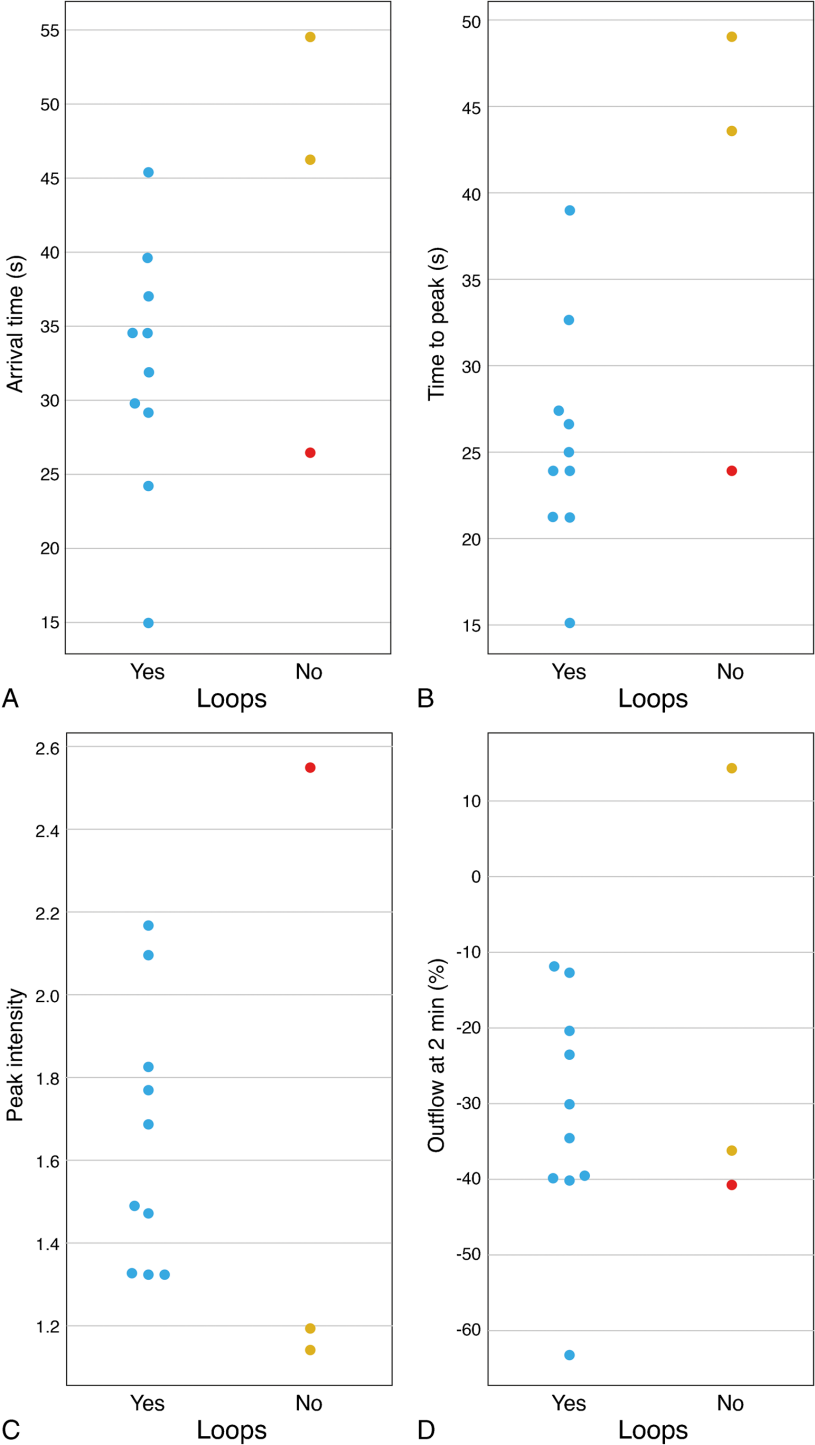
Comparison between the presence of loops (extracellular matrix patterns “vascular loops”) in histopathology and the perfusion parameters. **A**–**D**. Scatterplots showing the relation between the presence of loops in histopathology and AT (**A**), TTP (**B**), PI (**C**), and OP2,% (**D**). Notice one UM without loops that, probably due its high vascularization, had perfusion values similar to the ones of UM with loops (red dots)

UM with loops tended to have a shorter arrival time, a shorter time to peak, and higher peak intensities. The outflow percentage at 2 min does not differ much in tumors with or without loops (Figure 9). There was however one UM without loops which perfusion values were similar to the ones of UM with loops. This concerned a very inflammatory and highly vascularized tumor, which probably accounted for its perfusion results.

## Discussion

Imaging UM with MR requires a dedicated eye protocol consisting of 2D MS TSE sequences, which are indispensable for delineating tumor boundaries; 3D TSE sequences, which allow retrospective reformatting in all directions and 3D reconstructions, and are essential to assess tumor geometry and accurate measurements; and DWI and PWI sequences, which aid in the differential diagnosis, and potentially, provide prognostic information, predict treatment response, and permit earlier assessment of tumor response to radiotherapy than US [3, 8, 11, 14–18].

UM arises in the uveal tract. Ninety percent of the UM were described to arise in the choroid, 7% in the ciliary body, and the remaining in the iris [4]. However, while in small tumors the epicenter of the lesion clearly points to the origin of a UM, in larger UM, which involve the choroid as well as the ciliary body, it can be difficult to determine the primary site of the tumor. Therefore, in our study, 38% of UM were classified as choroidal/ciliary body tumors.

In one series with 200 UM, UM were reported to have a dome shape in 38%, a mushroom shape in 36%, and a flat-lentiform shape in 27% [19]. It is important to be aware that the limits of flat tumors are difficult to determine with both US and MRI. Moreover, the radiotherapy planning of UM with complex tumor shapes, such as some tumors with a mushroom configuration, is more difficult and deserves special attention [20]. The mushroom configuration is associated with breaks in Bruch’s membrane [11, 21]. Our study was consistent; we showed the importance of multiplanar reconstructions with MRI on recognizing the mushroom configuration of UM and noticed a mushroom configuration mainly in large tumors.

The amount of pigmentation in UM is variable, and although pronounced pigmentation has been associated with a less favorable prognosis [19], the accurate prognostic significance of pigmentation warrants further analysis [22]. When pigmented, the distribution of melanin within the tumor can be either homo- or heterogeneous [11, 19]. When heterogeneous, frequently two different tumor components, with a different melanin content, exist and the tumor is called bipartite. Fundoscopy can only evaluate the visible superficial ventral part of the tumors [11, 19], being not representative in case of heterogeneous UM, and assessment of tumor pigmentation may be difficult in case of retinal detachment. MRI has the advantage that it enables assessment of the distribution of the signal intensity within the whole tumor [19]. We showed that the signal intensity on T1-WI significantly correlates with tumor pigmentation, which is largely consistent with Lemke et al. [19]. T1-hyperintense UM were always moderately or strongly pigmented on histopathology, while T1-hypointense UM were either pigmented or non-pigmented.

We used a non-EPI DWI TSE technique for the orbit, as it is less susceptible to the present magnetic field inhomogeneities [8], and we used a small slice thickness (2.4 mm) in order to reduce partial-volume averaging effects to a minimum. In the present study, we found a mean ADC of 1.16 × 10^−3^ mm^2^/s, which is consistent with the reports from Foti et al. [3], Kamrava et al. [15], and Sepahdari et al. [23]. The study from Erb-Eigner et al*.* showed a lower mean ADC of 0.89 × 10^−3^ mm^2^/s [24], due to the larger size of the UM (mean diameter of 14 mm), or due to the potential overall lower ADC value of their protocol (mean ADC of the vitreous of 2.76 × 10^−3^ mm^2^/s) [24].

Dynamic contrast-enhanced MR imaging confirms that the tumor is enhancing. That is important in some T1-hyperintense UM where one might doubt whether there is enhancement from the evaluation of the pre- and post-contrast series [8]. The time-intensity curve can be qualitatively analyzed in 3D or 2D. In homogeneous tumors, the 3D evaluation performs better as it provides information on the entire UM. However, in heterogeneous tumors, especially in bipartite tumors, different tumor components may have different TICs and therefore a separate evaluation of both tumor components is advised. Overall, two-thirds of the UM had a wash-out curve, with a plateau curve in the other one-third, consistent with the reports from Yuan et al*.* [14] and Li et al. [25]. We noticed that both an increase of the tumor prominence and a decrease of the ADC favor a wash-out TIC. We found a mean peak intensity of UM of 1.62, consistent with Buerk et al*.* [26].

MRI potentially plays a pivotal role at the differential diagnosis [27]. Benign ocular lesions are expected to have higher ADCs [16] and mostly a progressive or plateau TIC at DCE, although functional evaluation of choroidal nevi can still be hampered by their very small size. In other intraocular malignant lesions, such as metastases and lymphomas, the main clues for the differential diagnosis are the lesion number, configuration, and signal intensity. For example, in the study from Lemke et al*.* with 200 UM, no tumor had a flat-placoid shape [27], which is the predominant shape of ocular metastases.

Assessment of tumor size is essential for the choice of treatment modality and planning of radiotherapy. The most accurate way to measure TP and LBD is the use of multiplanar reconstructions of isotropic 3D MR sequences [8]. Measuring the tumor’s LBD with MRI is only limited in case of flat tumors, with a tendency to underestimate the size due to unclear tumor margins. In comparison to MR, US tends to overestimate tumor size, likely due to oblique measurements whenever the transducer cannot be positioned perpendicular to the tumor [2, 11, 28].

Extraocular growth is associated with an increased rate of orbital recurrence and worse survival [29, 30]. It should be diagnosed in order to be taken into account in the treatment plan. We found extrascleral extension in 7% of the UM, consistent with current literature [27, 29–31]. Interestingly, in the present study, all observed extrascleral extensions occurred in small tumors with a maximum prominence of 4.8 mm, although in general, it occurs more commonly in medium or large tumors [29, 32]. Extrascleral extension often develops along scleral canals, via perivascular or perineural invasion. Therefore, the scleral underlying the tumor does not need to be invaded and the extrascleral tumor can even be located further away from the intraocular tumor [29, 30]. We had no false-positive cases with MRI, and one false-negative case where the extrascleral extension was 1.1 mm and anteriorly located. Diagnosing extrascleral extension with MRI is more challenging at the bulbar insertion of the extraocular muscles and anteriorly adjacent to the enhancing eyelid structures, although the latter extensions are usually readily visible clinically. Venous ectasia is another pitfall but it is more serpiginous in configuration [32]. MRI has been reported to be more accurate and judged with more confidence than US in the diagnosis of extrascleral extension [32]. In our study, from the three extrascleral extensions seen with MRI, US missed one case, which corresponded to the case of optic nerve invasion.

In one series with 1527 enucleated eyes due to UM, scleral invasion was histologically present in 56% of the cases [31]. An irregular inner contour and slight enhancement of the sclera adjacent to a UM on MRI should prompt the suspicion of scleral invasion. However, contrarily to what has been previously reported [33], scleral invasion is difficult to diagnose with MRI. All cases of histopathologic proven scleral invasion were missed on MRI and only in 20%, it was possible to see it retrospectively.

Optic nerve invasion is graded as prelaminar, laminar, or postlaminar, if tumor cells are present in the optic nerve head or in or beyond the lamina cribrosa, respectively [34]. In the present study, MRI demonstrated or ruled out postlaminar optic nerve invasion reliably in all cases. Optic nerve invasion needs to be assessed because its presence is a contraindication for ruthenium brachytherapy, and especially when it occurs postlaminar, it is associated with a propensity to orbital recurrence and poor prognosis [34].

We observed retinal detachment on MRI in 62% of the cases, more prevalent in tumors with a larger prominence and larger diameter, which is consistent with the literature [19, 27, 32]. It is important to differentiate tumor tissue from retinal detachment, especially in order to not overestimate the LBD. On MRI, retinal detachment does not enhance, there is no diffusion restriction (except when hemorrhagic), and it has a lentiform shape and a typical V shape with the vertex at the optic nerve [8, 19, 24]. In our study, MRI performed better than US in depicting RD.

It would be desirable to have noninvasive markers that may predict a tumor’s response to therapy and provide prognostic information. A biopsy is an invasive procedure and may not always be representative because chromosomal aberrations can be heterogeneous across the tumor [35, 36] and they seem to change with time. DWI and PWI are potentially useful but have hardly been evaluated. It is the current belief that the genetic profile governs prognosis and not the treatment modality.

Both the presence of monosomy 3 [22, 37, 38] and of extracellular matrix patterns “loops” [39–44] are important determinants of poor prognosis and they frequently coexist. All our tumors with loops had monosomy 3 and vice versa, consistent with the literature where monosomy 3 is detected in 67% of tumors with loops [40]. The study from Kamrava et al. found a significant correlation between monosomy 3 and perfusion values such as higher *k*^trans^ and *v*_e_ [15]. Interestingly, in our study, it seems that UM with loops also tend to have different perfusion values than UM without loops, such as a shorter time to peak and a bigger peak intensity, consistent with the fact that extracellular matrix patterns are pseudovascular channels, seeming to conduct plasma and sometimes blood. Because peak intensity and *k*^trans^ are related, our results add an extra meaning to the association described by Kamrava et al. [15], corroborating the promising value of PWI in recognizing UM with a poor prognosis.

## Conclusion

In this study, we comprehensively assessed the anatomical and functional MRI characteristics of UM. Knowing the MRI characteristics of UM is important in order to confirm the diagnosis of UM and to differentiate UM from other intra-ocular lesions and because it has implications for treatment planning. MRI is a good imaging technique for the assessment of size, shape, and local extent of UM, seeming more accurate than US, and only being limited in case of flat tumors and for the diagnosis of scleral invasion. The promising value of PWI on the identification of UM at higher risk of metastasis needs further investigation, as it could serve as a substitute for histopathology in patients that undergo an eye-sparing treatment.

## Supplementary information

ESM 1 (DOCX 24 kb)

ESM 2 (PNG 165 kb)

High resolution (TIF 2518 kb)

ESM 3 (PNG 188 kb)

High resolution (TIF 2116 kb)
