# Supplementary material for: MR imaging characteristics of uveal melanoma with histopathological validation
Source: Neuroradiology. 2021 Oct 31;64(1):171–84. doi: 10.1007/s00234-021-02825-5 (PMC8724164; doi:10.1007/s00234-021-02825-5)
Supplement: Supplementary file 1 — (DOCX 24 kb) [file 234_2021_2825_MOESM1_ESM.docx]

| **Patients** | **Age** | **Sex** | **Eye** | **c T-Stage** | **Treatment** | **Histology** |
| --- | --- | --- | --- | --- | --- | --- |
| **1** | 80 | female | OS | T3b | enucleation | melanoma |
| **2** | 73 | female | OD | T1c | enucleation | melanoma |
| **3** | 62 | male | OD | T4b | enucleation | melanoma |
| **4** | 60 | male | OD | T3a | enucleation | melanoma |
| **5** | 75 | male | OD | T4b | enucleation | melanoma |
| **6** | 47 | female | OD | T2a | RuPB |  |
| **7** | 67 | female | OD | T2a | PBT |  |
| **8** | 64 | female | OS | T3b | PBT |  |
| **9** | 46 | male | OS | T4b | enucleation | melanoma |
| **10** | 53 | male | OS | T1c | enucleation | melanoma |
| **11** | 45 | female | OD | T2b | PBT |  |
| **12** | 52 | male | OD | T3b | enucleation | melanoma |
| **13** | 70 | male | OD | T1a | RuPB |  |
| **14** | 31 | male | OD | T1a | RuPB |  |
| **15** | 66 | male | OS | T2a | RuPB |  |
| **16** | 64 | female | OD | T3b | enucleation | melanoma |
| **17** | 74 | male | OD | T2b | RuPB |  |
| **18** | 81 | male | OD | T3b | enucleation | melanoma |
| **19** | 48 | male | OD | T2a | enucleation | melanoma |
| **20** | 65 | male | OD | T3b | PBT |  |
| **21** | 66 | male | OS | T1a | PBT |  |
| **22** | 75 | male | OD | T1a | RuPB |  |
| **23** | 90 | male | OD | T1a | enucleation | melanoma |
| **24** | 24 | female | OS | T2c | RuPB |  |
| **25** | 59 | male | OD | T3a | RuPB |  |
| **26** | 59 | female | OD | T1a | RuPB |  |
| **27** | 59 | male | OD | T1a | RuPB |  |
| **28** | 70 | male | OS | T2b | RuPB | melanoma |
| **29** | 76 | male | OD | T1a | RuPB |  |
| **30** | 82 | male | OS | T3b | enucleation | melanoma |
| **31** | 61 | male | OD | T3a | enucleation | melanoma |
| **32** | 61 | male | OS | T1a | RuPB |  |
| **33** | 68 | male | OS | T2b | RuPB |  |
| **34** | 68 | male | OS | T3b | RuPB |  |
| **35** | 71 | male | OD | T3a | PBT |  |
| **36** | 73 | male | OD | T1a | RuPB |  |
| **37** | 38 | female | OD | T2a | RuPB |  |
| **38** | 51 | female | OS | T2a | RuPB |  |
| **39** | 55 | female | OS | T2a | RuPB |  |
| **40** | 76 | male | OS | T2a | PBT |  |
| **41** | 65 | male | OS | T3b | PBT |  |
| **42** | 63 | female | OS | T4e | PBT |  |

**Table 1.**

Patients’ data regarding age, sex, eye involved, clinical T-Stage (TNM Eighth edition/AJCC Eighth Edition), treatment and, in case of enucleation and/or biopsy, histology.

OD – oculus dexter

OS – oculus sinister

c T-Stage - clinical T-Stage

RuPB – Ruthenium plaque brachytherapy

PBT – Proton beam therapy

| **Patients** | **Cell Type** | **BAP1** | **Monosomy 3** | **Loops** |
| --- | --- | --- | --- | --- |
| **1** | spindle | loss | yes | yes |
| **2** | spindle | no loss | no | - |
| **3** | mixed | loss | yes | yes |
| **4** | epithelioid | loss | yes | yes |
| **5** | spindle | loss | yes | yes |
| **9** | epithelioid | no loss | no | no |
| **10** | spindle | loss | yes | yes |
| **12** | spindle | no loss | yes | yes |
| **16** | epithelioid | loss | yes | yes |
| **18** | mixed | no loss | yes | yes |
| **19** | spindle | no loss | no | no |
| **23** | spindle | no loss | no | no |
| **30** | mixed | loss | yes | yes |
| **31** | mixed | loss | yes | yes |

**Table 2**

Available histopathological and genetic data of the enucleated eyes.
