# Supplementary figures and images for: MR imaging characteristics of uveal melanoma with histopathological validation
Source: Neuroradiology. 2021 Oct 31;64(1):171–84. doi: 10.1007/s00234-021-02825-5 (PMC8724164; doi:10.1007/s00234-021-02825-5)

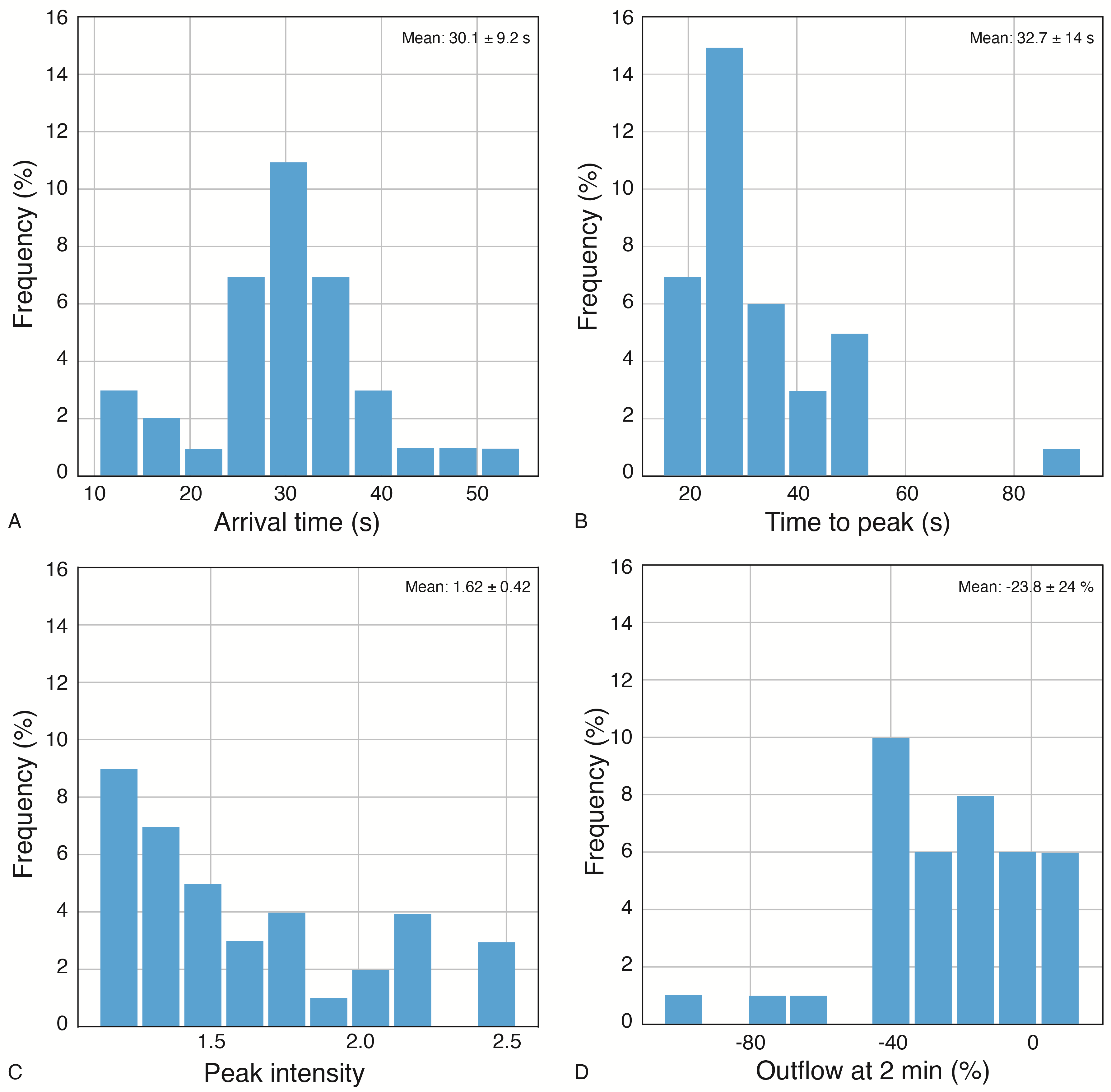

Supplement: Supplementary file 2 — (PNG 165 kb) [file 234_2021_2825_Fig10_ESM.png]

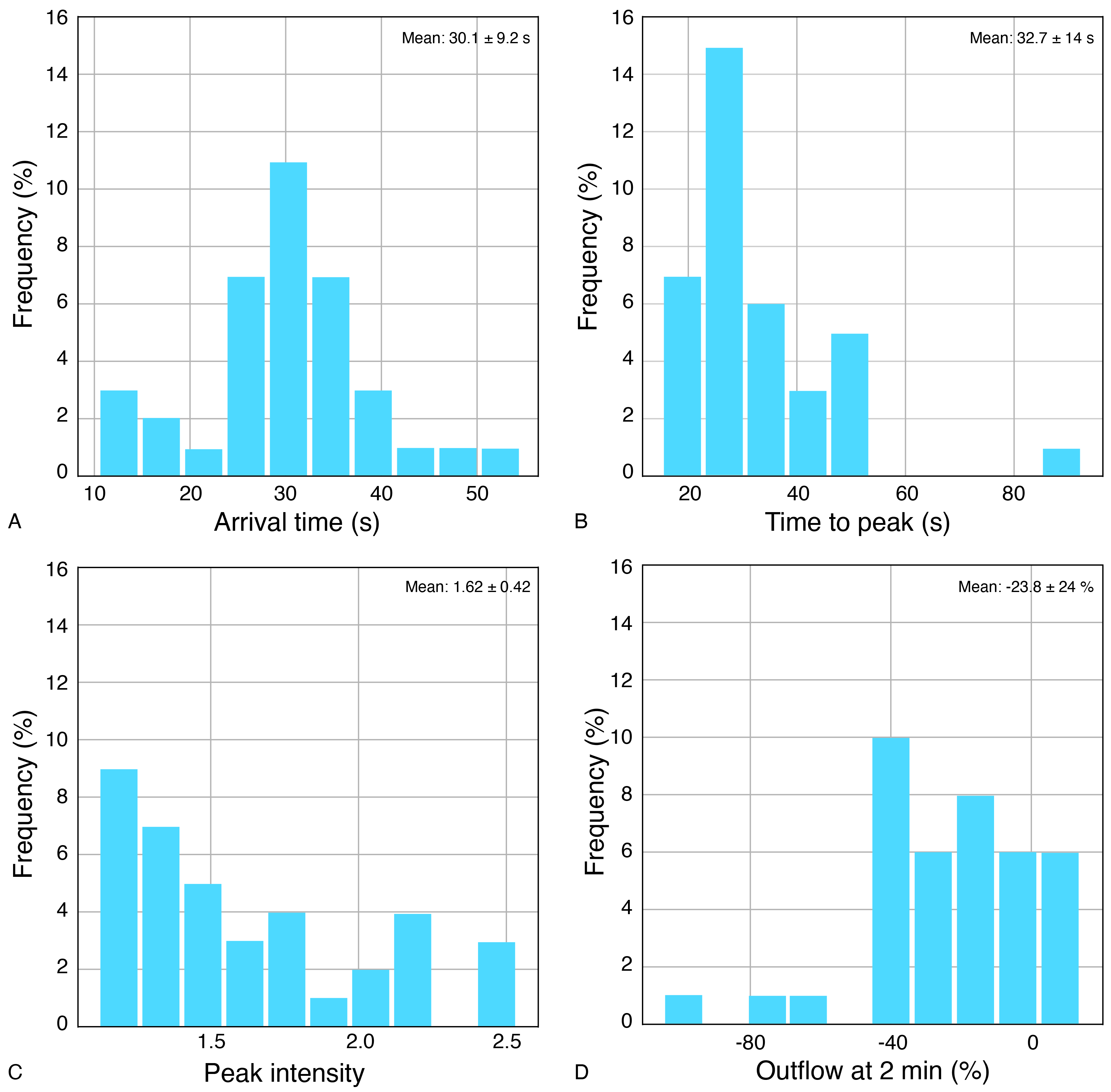

Supplement: Supplementary file 3 — High resolution (TIF 2518 kb) [file 234_2021_2825_MOESM2_ESM.tif]

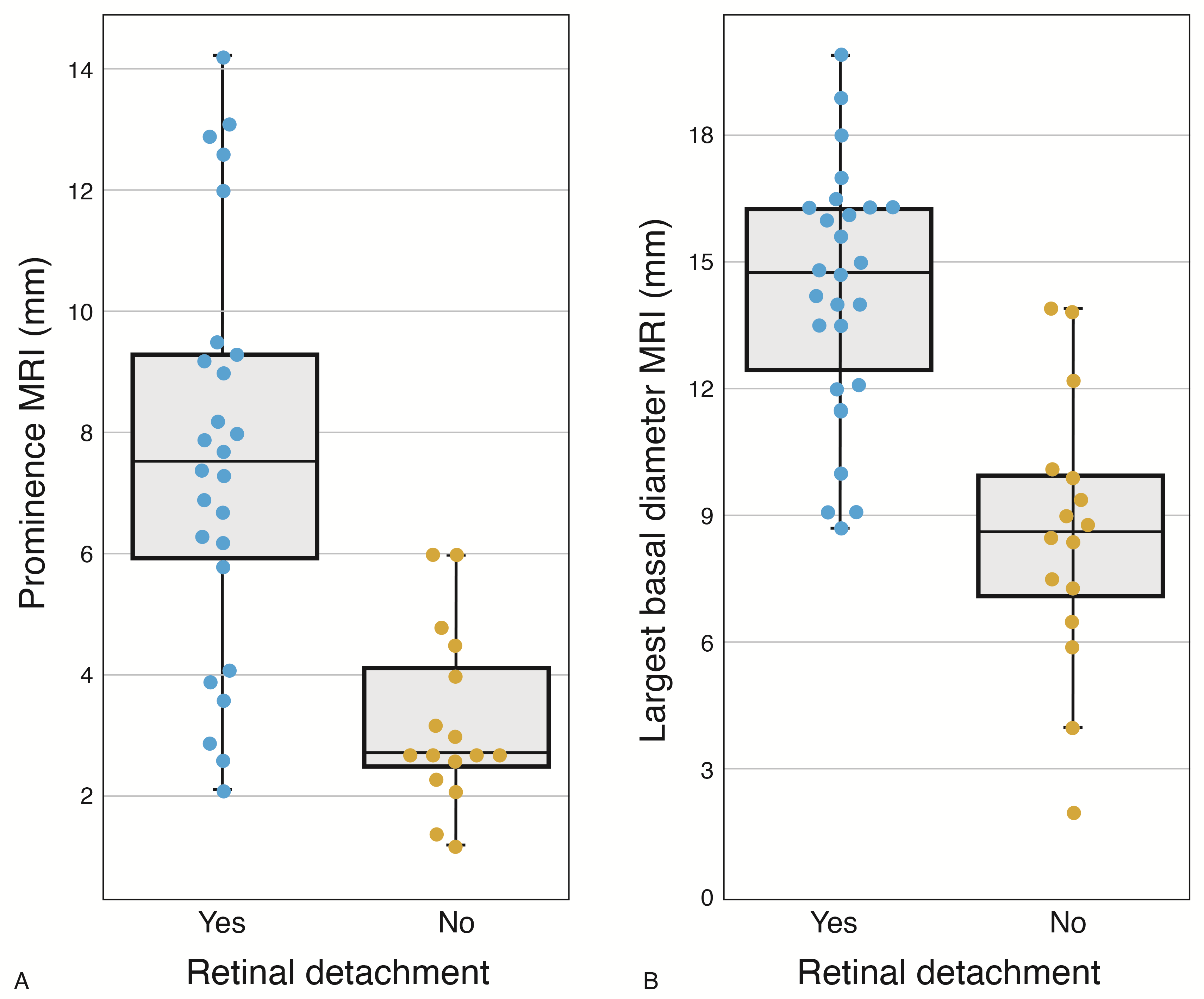

Supplement: Supplementary file 4 — (PNG 188 kb) [file 234_2021_2825_Fig11_ESM.png]

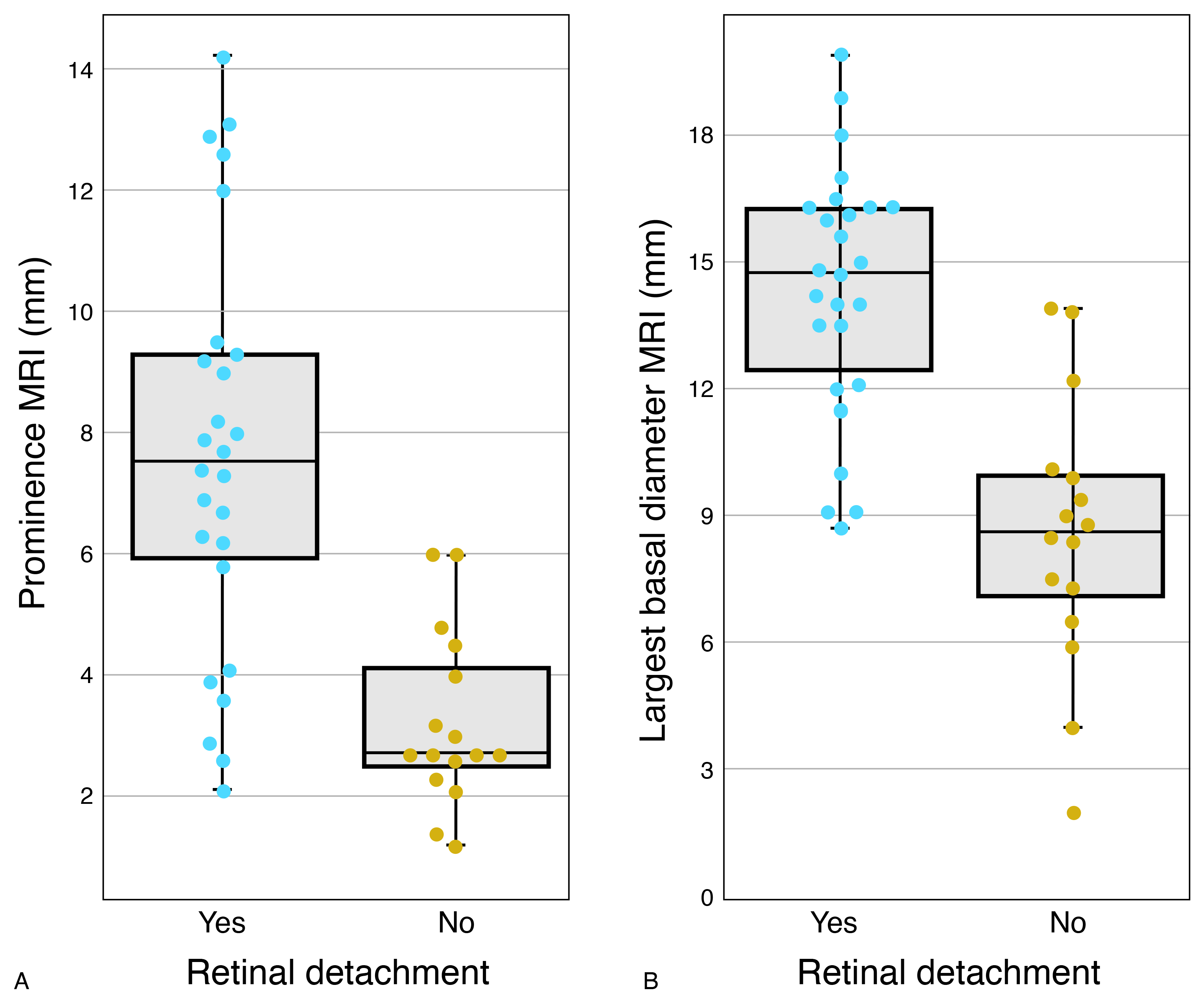

Supplement: Supplementary file 5 — High resolution (TIF 2116 kb) [file 234_2021_2825_MOESM3_ESM.tif]
